# Treatment of Dysmenorrhea by Hypodermic Injections of Phenic Acid

**Published:** 1890-03

**Authors:** 


TREATMENT OF DYSMENORRHEA BY HYPODERMIC INJECTIONS OF PHENIC ACID.—The solution recommended by Dr. Chéron, Paris, was as follows:

| | |
|---|---|
| Phenic Acid . . . . . . . . . . . . . . . . . . . . . | 2 grammes. |
| Aquæ destillat. . . . . . . . . . . . . . . . . . . . | 100 grammes. |

The injections are made in the abdominal parietes—or in the sacro-lumbar region—according to the location of the pain. An injection of five grammes is made at the beginning of the menstrual flow, and may be repeated two or three times daily. The following month injections of ten grammes, daily, are recommended, the week preceding the flow.—*Gazette de Gynécologie.*

